# Conditional genotype analysis: detecting secondary disease loci in linkage disequilibrium with a primary disease locus

**DOI:** 10.1186/1753-6561-1-s1-s163

**Published:** 2007-12-18

**Authors:** Glenys Thomson, Ana Maria Valdes

**Affiliations:** 1 Department of Integrative Biology, University of California, 3060 VLSB MC#3140, Berkeley, California 94720, USA

## Abstract

A number of autoimmune and other diseases have well established HLA associations; in many cases there is strong evidence for the direct involvement of the HLA class II peptide-presenting antigens, e.g., HLA DR-DQ for type 1 diabetes (T1D) and HLA-DR for rheumatoid arthritis (RA). The involvement of additional HLA region genes in the disease process is implicated in these diseases. We have developed a model-free approach to detect these additional disease genes using genotype data; the conditional genotype method (CGM) and overall conditional genotype method (OCGM) use all patient and control data and do not require haplotype estimation. Genotypes at marker genes in the HLA region are stratified and their expected values are determined in a way that removes the effects of linkage disequilibrium (LD) with the peptide-presenting HLA genes directly involved in the disease. A statistic has been developed under the null hypothesis of no additional disease genes in the HLA region for the OCGM method and was applied to the Genetic Analysis Workshop 15 simulated data set of Problem 3, which mimics RA (answers were known). In addition to the primary effect of the HLA DR locus, the effects of the other two HLA region simulated genes involved in disease were detected (gene C, 0 cM from DR, increases RA risk only in women; and gene D, 5.12 cM from DR, rare allele increases RA risk five-fold). No false negatives were found. Power calculations were performed.

## Background

Features of complex genetic diseases showing HLA associations, such as incomplete penetrance, multiple disease-predisposing loci both within and outside the HLA region, heterogeneity, and interaction effects, make determination of their genetic basis more difficult than for traits showing classic monogenic Mendelian inheritance. Nonetheless, the direct involvement of HLA class II, and in some cases class I, genes in the disease process has been well documented for a number of complex diseases. Many of these diseases are of autoimmune origin, but cancers, infectious, and other diseases are also included. The issue of contributions to these diseases by other HLA region genes has been of interest for a number of years. Many reports of other associations within the HLA region have appeared in the literature. However, in many of these studies it has been difficult to determine whether an additional HLA region gene is involved in disease, versus the association's reflecting linkage disequilibrium (LD) with the peptide-presenting HLA molecules directly involved in disease.

A number of analytic strategies have been developed to remove the effects of LD with the peptide-presenting HLA genes directly involved in the disease. These include matched genotype strategies [1], and for family-based data the homozygous parent transmission disequilibrium test (HPTDT) [2] and the homozygous parent linkage test (HPLT) for affected sib pairs [3]. These three approaches only use a subset of the patient and control data. The haplotype method (henceforth referred to as the conditional haplotype method (CHM)) [4-6] and the conditional extended TDT (CETDT) [7,8] use more of the data, but haplotypes need to be estimated. The logic of the CHM starts with the assumption that all HLA genes directly involved in disease susceptibility have been identified, e.g., HLA DR in RA. We then examine the *relative *frequencies of alleles at marker loci (e.g., single-nucleotide polymorphisms (SNPs), microsatellite markers, or other classical HLA loci) located on *specific haplotypes *stratified (to remove the effects of LD) by HLA DR in the case of RA, e.g., the high risk DRB1*0401 allele. Under the null hypothesis, the *relative *frequencies of the marker alleles on these stratified haplotypes should be the same in cases and controls. While fit to these expectations does not exclude the possibility that other genes in the HLA complex are involved in disease, lack of fit unequivocally shows that all disease-predisposing genes in the region have *not *been identified (provided that population stratification effects, for example, have not produced spurious results). The CHM tests for effects on individual haplotypes at the primary disease predisposing locus. Thomson [9] extended the CHM approach with development of a method to test for additional genetic effects *over all haplotypes*, henceforth referred to as the overall conditional haplotype method (OCHM). One advantage of the CHM, OCHM, and CETDT is that more of the data is used than with the matched genotype approach and the HPTDT and HPLT methods. However, care must be taken in all analyses and their interpretations with rare haplotypes and sparse cells.

Our aim in this study is to present a *conditional genotype approach *that tests for the presence of additional disease genes in a genetic region while accounting for LD with a primary disease gene. In the conditional genotype method (CGM), marker genotype frequencies are compared between cases and controls (AFBACs (affected family-based controls) in the case of family data [10,11]). Our concentration in this paper is on the overall conditional genotype method (OCGM) in which genotype frequencies at marker loci are considered over all genotypes at the primary disease locus (in fact only all common genotypes are considered). We have developed an appropriate test statistic and applied this method to the Genetic Analysis Workshop 15 (GAW15) Problem 3 data (with answers known).

## Methods

### Model

Let *A *be the primary disease locus of interest, with penetrance value *w*_*ij *_for genotype *A*_*i*_*A*_*j*_. Under the null hypothesis, the *B *locus is neutral with respect to disease and the *A *locus accounts for all disease risk in this genetic region. The frequencies of genotypes in controls at the *A *locus are denoted *f*_*c*_(*A*_*i*_*A*_*j*_) and *f*_*p*_(*A*_*i*_*A*_*j*_) for patients. The expected genotype frequencies among patients are given by:

*f*_*p*_(*A*_*i*_*A*_*j*_) *= w*_*ij*_*f*_*c*_(*A*_*i*_*A*_*j*_)*/T*,

where T is a normalizing factor. No assumption of Hardy Weinberg proportions in controls or patients is needed. Rewriting Eq. (1a), the relative penetrance values are estimated as follows:

*w*_*ij *_/*T = f*_*p*_(*A*_*i*_*A*_*j*_)*/f*_*c*_(*A*_*i*_*A*_*j*_).

Adding the second neutral locus *B*, the expected two-locus genotype frequencies under the null are given by:

exp *f*_*p*_(*A*_*i*_*A*_*j*_*B*_*k*_*B*_*l*_) = *w*_*ij *_*f*_*c*_(*A*_*i*_*A*_*j*_*B*_*k*_*B*_*l*_)/*T*,

and substituting from Eq. (1b) gives

exp *f*_*p*_(*A*_*i*_*A*_*j*_*B*_*k*_*B*_*l*_) = *f*_*c*_(*A*_*i*_*A*_*j*_*B*_*k*_*B*_*l*_) [*f*_*p*_(*A*_*i*_*A*_*j*_)*/f*_*c*_(*A*_*i*_*A*_*j*_)].

### Conditional genotype method (CGM)

For a given *A*_*i*_*A*_*j *_genotype, the term in [.] in Eq. (2b) is constant, so under the null that the *A *locus is the only disease-predisposing gene in the region, the relative genotype frequencies at the *B *locus should show no heterogeneity between patients and controls. We term this the conditional genotype method (CGM), with analogy to the conditional haplotype method (CHM) discussed above. In both cases, statistical testing can be done via a chi-square test of heterogeneity, with all the associated caveats therein, Fisher's exact test, or a resampling approach. We have not pursued this approach in the current analyses.

### Overall conditional genotype method (OCGM)

If the effect of an additional disease-predisposing gene in the region, e.g., the *B *gene or one in LD with it, is not specific to a particular haplotype or genotype at the primary disease locus *A*, then more power should be available by considering *B *locus genotypes combined over all *A *locus genotypes. In this case, the expected genotype frequencies at the *B *locus under the null hypothesis, assuming that *A *(disease locus) segregates for *m *alleles and *B *(putative neutral locus) for *n *alleles, are given by:

$$\exp f_{p}(B_{h}B_{k})=\sum_{i=1}^{m}\sum_{j=i}^{m}\frac{f_{p}(A_{i}A_{j})}{f_{c}(A_{i}A_{j})}f_{c}(A_{i}A_{j}B_{h}B_{k}).$$

The question of statistical testing then arises. Application of a standard test of homogeneity of the *B *genotype observed (obs) and expected (exp) numbers does not give a chi-square distribution, in fact the distribution is exponential. This is due to the use of the *fp*(*A*_*i*_*A*_*j*_)*/fc*(*A*_*i*_*A*_*j*_) ratio in the estimation of expected values. This observation led to the test statistic suggested below [12]. Note also that the use of low-frequency control genotype frequencies (*fc*(*A*_*i*_*A*_*j*_)) could be problematic notwithstanding and these should be left out of any analyses, although this was not necessary in the current analysis of simulated data.

We propose the following test statistic when comparing exp *f*_*p*_(*A*_*i*_*A*_*j*_*B*_*k*_*B*_*l*_) to the observed value *f*_*p*_(*A*_*i*_*A*_*j*_*B*_*k*_*B*_*l*_) in our statistical testing. For consistency we will refer to the latter as obs *f*_*p*_(*A*_*i*_*A*_*j*_*B*_*k*_*B*_*l*_):

$$\chi_{2df}^{2}=\frac{Np+Nc}{2}\sum_{h,k}c_{hk}$$

where:

$$c_{hk}=\frac{{\left(\exp f_{p}(B_{h}B_{k})-obs\;f_{p}(B_{h}B_{k})\right)}^{2}}{4(\exp f_{p}(B_{h}B_{k})+obs\;f_{p}(B_{h}B_{k}))},$$

and exp *f*_*p*_(*B*_*h*_*B*_*k*_) is given by Eq. (3). Note that no LD or haplotype estimates are needed. With respect to analyses of the GAW15 Problem 3 data we discuss power issues in the Results section below and show that this test statistic has the nominal *p*-value for markers with no effect on disease and not in LD with loci C and D.

## Results

**Figure 1:**
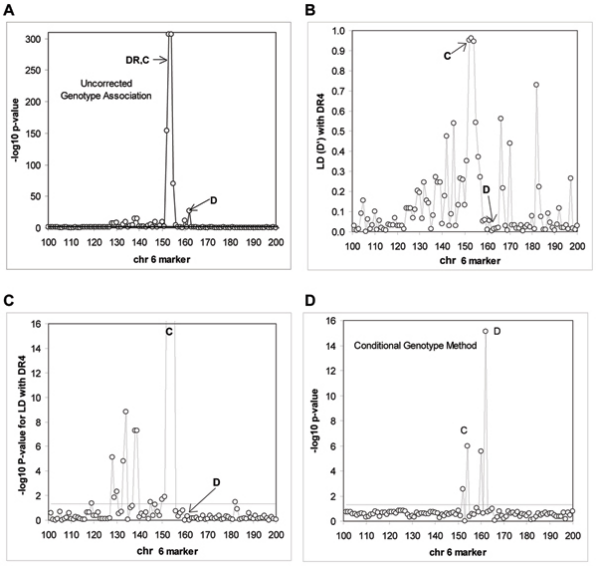
**Genetic association, linkage disequilibrium and application of conditional genotype analysis to chromosome 6 simulated data**. A, Genotype associations of HLA region (chromosome 6) SNPs; B, linkage disequilibrium of SNPs 100 to 200 with the HLA DR4 allele; C, physical distribution of significant LD with chromosome 6 SNPs; D, application of the overall conditional genotype method (OCGM) to the HLA region data. Results refer to the average of 50 simulation replicates.

On the simulated RA-like Problem 3 data we used one affected sib from all families (*n *= 1500) and all unrelated controls (*n *= 2000) and studied 674 SNPs on chromosome 6. First we tested for genotype associations of each SNP with disease, and found significant effects around the HLA region (Figure 1A); however the DR and C locus effects were intertwined. We then concentrated on SNPs 100–200 (the HLA region) in 50 replicates. A number of SNPs showed associations, and these correlated with estimated LD with the HLA DR4 allele simulated in the data (allele 3 of the simulation) (Figure 1B and 1C). Significant LD with DR4 extends from SNP 119 to 183, although except for these two extremes, the significant LD falls between SNPs 129 to 155.

Applying the OCGM to the data, the effects of Locus C and Locus D, additional to those of HLA-DR were found (Figure 1D). The statistical power to detect the D locus was higher, given that we only looked at the complete sample without stratifying by gender with respect to Locus C. However, for Loci C and D loci the power was greater than 80% at the *p *< 0.05 level and the type I error observed for markers outside the main LD region with DR, C, or D are consistent with theoretical expectations (Table 1). We note that the type I error for markers 100 to 130 is slightly higher than the nominal 5% and 1% because a few markers are in significant LD with DR.

**Table 1:** Statistical power to detect Locus C and D effects using the overall conditional genotype method (OCGM) based on 50 simulation replicates

Marker^a^	Disease locus	*p *< 0.05	*p *< 0.01
100–139	none	6.0%	2.1%
152	C	24.0%	4.0%
153	DR	0.0%	0.0%
154	C	98.0%	72.0%
160	D	72.0%	50.0%
162	D	96.0%	94.0%
170–200	none	5.3%	1.2%

^a^The average for markers number 100–139 and 170–200 is also shown.

## Conclusion

We have developed a simple but powerful approach using genotypes (the conditional genotype method (CGM) and overall conditional genotype method (OCGM) method) to detect association of a secondary locus in LD with a major disease locus that is model free and does not require estimation of haplotype frequencies. Even with strong LD as seen with the DR and C loci in this data set, we were able to detect the effect of the C locus after allowing for the effect of the primary DR locus. This was possible even though D' = 1 for these two loci, as both alleles C and c occur with allele DRX. In this case DRX refers to all alleles that are not DR4 and not DR1, namely, allele 1 of the simulation for the DR locus. The allele frequencies of these two haplotypes were 0.15 and 0.50, respectively. This allowed discrimination between risk effects of the genotypes comprising these two haplotypes, and hence detection of the C locus effect once the primary effect of the DR locus was taken into account. The only situation in which the CGM and OCGM, as well as other methods, would not be useful is when there is an absolute 100% correlation between all alleles at both loci. In such cases, cross ethnic studies may be useful in that the LD pattern may differ and not show 100% correlation.

One could also take the observed (obs) and expected (exp) *fp*(*B*_*h*_*B*_*k*_) values from Eq. 3 and consider the observed and expected allele frequencies at the *B *locus under the null; obtained simply by the method of allele (gene) counting. In effect, this is the same as the overall conditional haplotype method (OCHM) [9] but the genotype approach obviates the need to estimate LD values in controls between the *A *and *B *genes as proposed in its initial implementation. Another difference is that whereas the genotype-based implementation uses ratios of *A *locus genotype frequencies in patients versus controls, the haplotype-based implementation as originally proposed uses allele frequencies. The effect of this on type I and type II errors is not known and further work would be required to determine the appropriate test statistic for the OCHM or a resampling approach could be applied. Thus, we have shown theoretically how allele frequency data can also be tested without resorting to estimation of haplotype frequencies using the OCHM. The OCHM has not been applied in this current research.

Our theory, applied to GAW15 Problem 3 data, and power analyses of the OCGM, as well as discussion of the CGM, CHM, and OCHM, show the utility of all these approaches for detecting additional disease genes in LD with a primary disease gene in a genetic region. Additionally, all of these methods are useful in detecting primary disease-predisposing genes in a region if they are not known. Also, they can be used in across-genome studies to detect the effects of other disease genes that have risk effects that are not strictly multiplicative with a primary disease gene under study, and hence the effects may be easier to detect in samples stratified by the genotypes of the primary disease gene.

## Competing interests

The author(s) declare that they have no competing interests.
